# A Methodology for Classifying Root Causes of Outbreaks of Legionnaires’ Disease: Deficiencies in Environmental Control and Water Management

**DOI:** 10.3390/microorganisms9010089

**Published:** 2021-01-01

**Authors:** Benjamin R. Clopper, Jasen M. Kunz, Simone W. Salandy, Jessica C. Smith, Brian C. Hubbard, John P. Sarisky

**Affiliations:** 1 Oak Ridge Institute for Science and Education, P.O. Box 117, Oak Ridge, TN 37830, USA; 2 Division of Environmental Health Science and Practice, National Center for Environmental Health, Centers for Disease Control and Prevention, Mailstop S106-5, 4770 Buford Highway, Atlanta, GA 30341, USA; vtb0@cdc.gov (S.W.S.); bnh5@cdc.gov (B.C.H.); zse1@cdc.gov (J.P.S.); 3 Division of Bacterial Diseases, National Center for Immunization and Respiratory Diseases, Centers for Disease Control and Prevention, Mailstop H24-6, 1600 Clifton Road, Atlanta, GA 30333, USA; lyd7@cdc.gov

**Keywords:** Legionnaires’ disease, water management, environmental health

## Abstract

We piloted a methodology for collecting and interpreting root cause—or environmental deficiency (ED)—information from Legionnaires’ disease (LD) outbreak investigation reports. The methodology included a classification framework to assess common failures observed in the implementation of water management programs (WMPs). We reviewed reports from fourteen CDC-led investigations between 1 January 2015 and 21 June 2019 to identify EDs associated with outbreaks of LD. We developed an abstraction guide to standardize data collection from outbreak reports and define relevant parameters. We categorized each ED according to three criteria: ED type, WMP-deficiency type, and source of deficiency. We calculated the prevalence of EDs among facilities and explored differences between facilities with and without WMPs. A majority of EDs identified (81%) were classified as process failures. Facilities with WMPs (*n* = 8) had lower prevalence of EDs attributed to plumbed devices (9.1%) and infrastructure design (0%) than facilities without WMPs (*n* = 6; 33.3% and 24.2%, respectively). About three quarters (72%) of LD cases and 81% of the fatalities in our sample originated at facilities without a WMP. This report highlights the importance of WMPs in preventing and mitigating outbreaks of LD. Building water system process management is a primary obstacle toward limiting the root causes of LD outbreaks. Greater emphasis on the documentation, verification, validation, and continuous program review steps will be important in maximizing the effectiveness of WMPs.

## 1. Introduction

Legionnaires’ disease (LD) is an often-severe pneumonia caused by an infection from bacteria of the genus *Legionella*. Overall, approximately 1 in 10 cases of LD is fatal [1,2,3], though higher fatality rates have been observed in healthcare settings [3,4]. The infection is transmitted through the inhalation of small water droplets that contain *Legionella*. Important risk factors for infection are history of smoking, increased age, immunocompromised health status, and other comorbid conditions [5,6]. Exposure to aerosolized *Legionella* can also cause a milder, self-limiting illness called Pontiac fever. Over 60 species [7,8] and 70 serogroups [9,10] of *Legionella* bacteria have been described. While at least twenty species are known to cause disease [10,11,12], *Legionella pneumophila* is the most important contributor to LD burden in the United States and Europe, where it represents an estimated 90% of total LD cases [7,8,10,12]. Of these cases, about 80% are due to *L. pneumophila* serogroup 1 [7,12].

Since the disease’s formal identification in 1976, the number of cases reported yearly has steadily increased [13]. The Centers for Disease Control and Prevention (CDC) reported 9933 cases of LD in 2018, which represents a nearly 900 percent increase since 2000 [13]. *Legionella* has accounted for a majority of potable-water-associated outbreaks reported to CDC in recent years, including 66% of all such outbreaks reported in 2011 and 2012 [14] and 57% of those reported in 2013 and 2014 [15]. Outbreaks of *Legionella* represented 61% of the waterborne outbreaks identified through the National Outbreak Reporting System in CDC’s latest report on data from 2017 [16]. Several theories have been proposed to explain the continued increase in LD prevalence, including improved surveillance, aging infrastructure (i.e., building water systems, heating, ventilation, and air-conditioning equipment), aging population, and a warming climate [5,6].

Legionnaires’ disease is a byproduct of the built environment [6]. Bacteria of the genus *Legionella* are common in freshwater ecosystems; however, ambient environmental conditions are typically insufficient to infect humans [11]. If introduced into a human-made water system, permissive temperature, stagnant water lines, and inadequate disinfectant can allow the bacteria to proliferate. Plumbed devices such as cooling towers, showerheads, faucets, and hot tubs can produce aerosols sufficient for transmitting the bacteria to susceptible hosts and have been implicated as an important source of *Legionella* exposure [5,9,11].

ASHRAE’s publication of Standard 188 in 2015, with update in 2018, emphasized the role of environmental health in the prevention of LD [17]. Standard 188 outlines industry best practices and calls for the implementation of comprehensive water management programs (WMPs) to reduce the burden of *Legionella* in complex building water systems. The importance of the environmental health standards and control measures set by ASHRAE-compliant WMPs has been recognized by the Centers for Medicare & Medicaid Services (CMS) as well as the Veterans Health Administration. As of 2017, both organizations have issued directives mandating the implementation of WMPs in certain healthcare facilities [18,19]. A recent review of international standards, guidelines, and protocols for preventing and controlling LD further reinforces the importance of environmental health-based control measures [20].

Environmental health (EH) perspectives and techniques are essential for identifying risk and controlling exposures to prevent LD [21]. Specialized knowledge of industrial hygiene and facility inspection practices is essential in determining how complex water systems treat, move, store, heat, and cool water throughout buildings. Environmental assessments are invaluable investigative tools that provide a systematic method of identifying and inspecting potential reservoirs and aerosol dispersal mechanisms during active outbreaks. Trained EH professionals use information gathered from environmental assessments to make recommendations for specific interventions to reduce exposures and break the cycle of transmission. Robust environmental sampling plans conducted in coordination with clinical surveillance and molecular techniques allow for collection, isolation, and sequencing of samples to identify an outbreak strain and track its remediation within a water system. The information gathered by EH professionals during LD outbreak investigations contains rich detail about the frequency and nature of water system management and maintenance deficiencies, but a standard protocol for collecting, interpreting, and storing this environmental information does not yet exist. Collecting, storing, and sharing data of this type are important steps toward mitigating environmental risks and targeting water management priorities [22].

In 2016, CDC classified deficiencies in the environmental control of complex water systems to highlight opportunities for LD prevention [5]. The researchers identified four types of environmental deficiencies (process failures, equipment failures, human errors, and unmanaged external changes) and recommended adoption of WMPs to prevent LD outbreaks [5]. Our work builds on the methodology outlined in 2016 by formalizing a protocol for the abstraction of environmental deficiency (ED) information observed during outbreaks of LD. In addition, our updated methodology includes a classification framework to assess common failures observed in the implementation of WMPs. This project represents the first review of WMP deficiencies observed in the field. Finally, this project lays a foundation for expanding use of the abstraction protocol beyond CDC to partners in state and local health departments.

## 2. Materials and Methods

State and local health departments regularly request assistance from CDC to conduct LD outbreak investigations. The type of assistance provided by CDC ranges from a limited, remote consultation with an LD subject matter expert, to a multimember team deployment for an onsite outbreak investigation that can last a week or longer. Since 2000, CDC has conducted 55 onsite LD outbreak investigations. During each response effort, the investigators document critical aspects of the outbreak including the epidemiology of the cases, findings from environmental assessments, environmental sampling results, and analysis of WMPs. As a result, these outbreak reports contain detailed information on the environmental conditions that have permitted the presence, growth, spread, or aerosolized distribution of *Legionella*.

The present update formalizes and expands the original ED categorization structure outlined in 2016 [5] and applies it to CDC-led LD investigations between 2015 and 2019. New additions to the categorization structure are three WMP-deficiency types (improper implementation, inadequate content, and no WMP in place) and a description of the source of each ED. We included reports from land-based LD outbreak investigations conducted onsite by CDC personnel between 1 January 2015 and 21 June 2019. We excluded cruise ship outbreak investigation reports because their water systems are managed differently from land-based water systems. We excluded outbreak investigation reports if the deficiencies in environmental control could not be linked to the associated outbreaks as direct contributors. We defined direct contribution as a clear epidemiologic signal that linked each LD case with exposure to a facility with one or more EDs identified in the outbreak investigation report.

**Figure 1:**
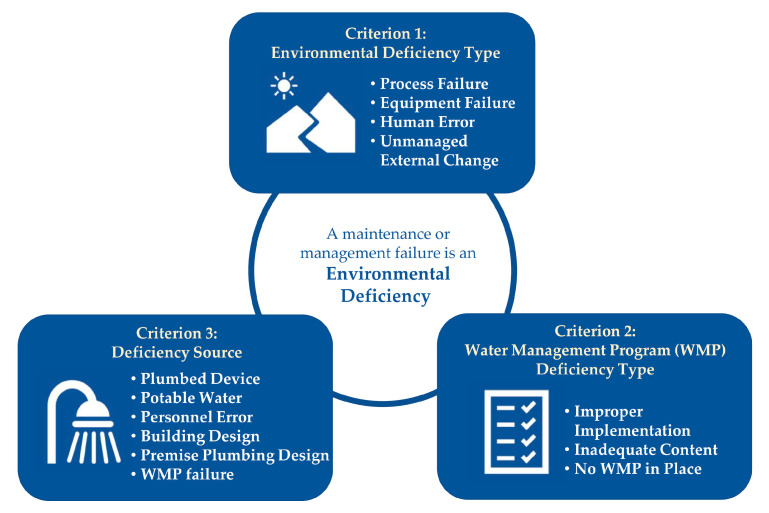
Each environmental deficiency is classified using three criteria.

The research team developed an abstraction guide to standardize data collection from the outbreak reports and define relevant parameters (see Supplementary Materials). We reviewed outbreak reports and sought to identify EDs, which we defined as maintenance or water management deficiencies that could permit the presence, growth, spread, or aerosolized distribution of *Legionella* in human-made water systems. We categorized each ED that we identified according to three criteria: ED type, WMP-deficiency type, and source of the ED. First, we classified these deficiencies using one or more environmental categories (Figure 1) as described by Garrison et al. [5]. We classified EDs as process failures when a specific process was missing or inadequate for preventing the growth or spread of *Legionella*, as human errors when a specific person did not perform as expected, as equipment failures when a specific piece of equipment did not operate as expected or was inadequate, and as unmanaged external changes when a specific adjustment was not made to account for events outside a building water system. The ED type categorizations were not mutually exclusive. An individual ED was classified with more than one ED type category if the descriptive information in the outbreak report provided evidence that more than one ED type category applied. Next, we linked each ED to one or more WMP-deficiency categorizations based on the aspect of water management that could have prevented the ED from occurring—inadequate WMP content, improper WMP implementation, or no WMP in place. Similar to our approach to ED type categorizations, we applied more than one WMP-deficiency type if the descriptive information in the outbreak report provided evidence for more than one WMP deficiency. We defined inadequate WMP content deficiencies as scenarios where an implemented WMP was missing one or more critical elements as described by CDC’s *Legionella* WMP Toolkit [23]. We defined improper WMP implementation deficiencies as scenarios where a WMP was sufficient as written, but improper implementation of the program created conditions in which one or more environmental failures occurred. We defined a no WMP in place deficiency as a scenario in which a facility lacked a WMP. We created the WMP-deficiency categories in order to frame the EDs that we observed in LD outbreaks from the perspective of water management-based solutions. Finally, we categorized the source of each individual ED based on the component of the water system in which the deficiency was identified, such as potable water disinfectant or a plumbed device.

Two authors abstracted the outbreak reports individually using qualitative analysis tools available in ATLAS.ti version 8.4.14.0 (ATLAS.ti Scientific Software Development GmbH, Berlin, Germany). The results of the individual abstractions were reviewed by authors with *Legionella*-specific expertise in environmental health and epidemiology. Any discrepant classifications from the round of individual abstractions were resolved through consultation with the subject matter experts.

We calculated the prevalence of deficiencies by ED type, WMP-deficiency type, and source across all fourteen outbreaks. We compared the prevalence of ED types, WMP deficiency-types, and sources of deficiencies in facilities with and without WMPs.

## 3. Results

CDC conducted 17 onsite investigations of LD outbreaks between 1 January 2015 and 21 June 2019. We included and abstracted a total of 14 investigation reports in this report. We excluded three community cluster investigations because there was insufficient epidemiologic evidence to implicate a specific building, water source, or plumbed device. Nearly three quarters of all outbreak investigations occurred in senior living/long-term care facilities (*n* = 5, 36%) or hospitals (*n* = 5, 36%). The remainder of the investigations occurred in hotels/resorts (*n* = 3, 21%) and one other venue (*n* = 1, 7%). Together, the investigation reports accounted for 125 confirmed cases of LD and 21 deaths (Table 1). The investigations identified 41 suspected, possible, or probable LD cases, as well as 12 cases of Pontiac fever. The median outbreak size was 5 cases, with a range of 1–46 cases. Seventy-two percent of the LD cases and 81% of the fatalities in our sample originated at facilities without a WMP in place. 

**Figure 2:**
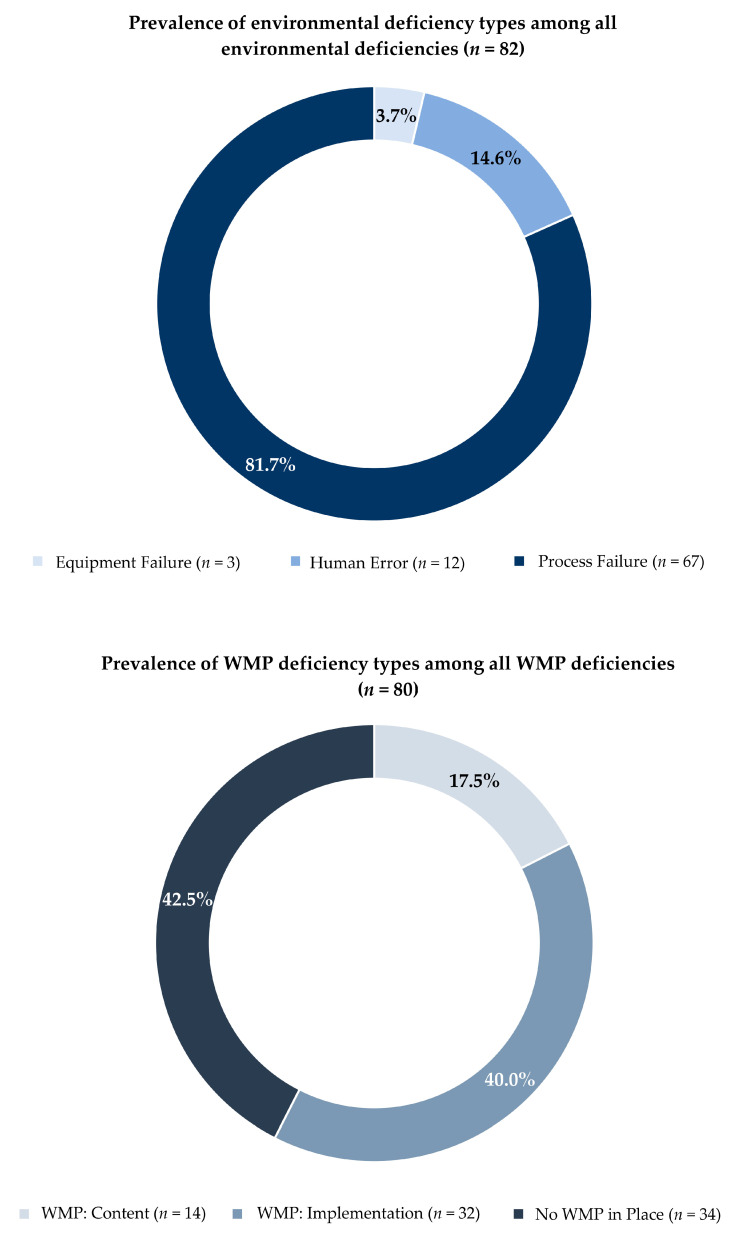
Prevalence of environmental deficiencies and water management program deficiencies, all sites.

A detailed account of every ED identified (*n* = 82) is included in Table S1 (see Supplementary Materials). In total, over 80% (*n* = 67) of the EDs identified in the investigation reports were classified as process failures (Figure 2). The remainder of the deficiencies were human errors (*n* = 12, 15%) and equipment failures (*n* = 3, 4%). No unmanaged external change EDs were identified in this report. Across thirteen investigation reports in our sample that contained a description of at least one ED, we found evidence of process failures in twelve (92%), human errors in nine (69%), and equipment failures in three (23%) reports. Over eighty percent of the total WMP deficiencies identified fell into two categories (Figure 2) —WMP implementation failure (*n* = 32, 40.0%) or no WMP in place (*n* = 34, 42.5%). About one in five WMP deficiencies was characterized as a WMP-content failure (*n* = 14, 17.5%). All investigation sites included in this review met criteria for recommending implementation of a WMP per CDC’s Legionella WMP Toolkit [23].

**Figure 3:**
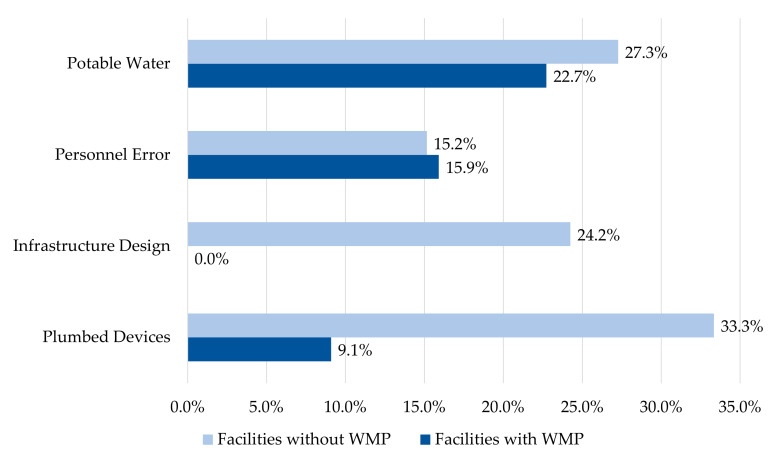
Prevalence of environmental deficiencies’ sources by water management status (*n* = 77).

In total, the most commonly identified sources of EDs were WMP failures (*n* = 23, 29.9%), potable water (*n* = 19, 24.7%), and plumbed devices (*n* = 15, 19.5%). When outbreak settings were divided into facilities with and without WMPs, the sources of EDs showed a distinct pattern (Figure 3). Compared to facilities without WMPs, facilities with WMPs had lower prevalence of deficiencies attributed to plumbed device sources (*n* = 4, 9.1% vs. *n* = 11, 33.3%) and infrastructure design sources (0% vs. *n* = 8, 24.2%), but similar prevalence of deficiencies attributed to potable water sources (with WMP: *n* = 10, 22.7%; without WMP: *n* = 9, 27.3%) and personnel error sources (with WMP: *n* = 7, 15.9%; without WMP: *n* = 5, 15.2%).

## 4. Discussion

Our data highlight the importance of comprehensive water management activities in safeguarding building water systems. The preponderance of process failures (such as permissive water temperatures and unlogged flushing events) over other ED types suggests that process management of building water systems represents the greatest challenge for controlling *Legionella*. This may be related to the size and complexity of WMPs for some facilities or to limited resources for development and refinement of WMPs. WMP implementation failures made up 70% of the WMP deficiencies in facilities under water management. This suggests that although the content of the WMPs at facilities evaluated in our sample was largely adequate for controlling the growth and spread of *Legionella*, interventions are needed to improve implementation of these WMPs. The high prevalence of implementation failures also serves as a reminder of the importance of the verification step (Is the WMP being implemented as designed?), validation step (Are each of the components of the WMP bringing about their intended effects?), documentation step (Are all WMP activities being recorded?), and continuous program review step, which are required by ASHRAE-compliant WMPs. Greater emphasis on these steps will be important in maximizing the effectiveness of WMPs as they become more widely adopted.

Nearly three quarters of the total LD cases and 8 in 10 fatalities in our sample originated at facilities without a WMP in place. A possible explanation of this observation is that facilities using a WMP may have an increased awareness of LD. These facilities might be better equipped to implement a quick and effective response once a confirmed case is identified. Staff at a facility with water management are more likely to be familiar with their building’s water system, and therefore, are better prepared to rapidly implement environmental controls when cases of disease or lapses in water management occur. In healthcare settings, increased awareness of LD might also improve clinical response during outbreaks, including case screening via urinary antigen test and collection of lower respiratory specimens for culture. Differences in the size of the facilities or in the underlying health status of a facility’s population might also have contributed to the discrepancy in cases and deaths we observed between facilities with managed and unmanaged water systems. It is important to note that water management procedures consistent with ASHRAE recommendations [17] could have addressed all 82 environmental deficiencies that we identified in this review.

The prevalence of different ED types was similar for facilities with and without WMPs. The ranked prevalence of process failures, human errors, and equipment failures is similar to the findings from the 2016 report [5]. Notably, the current report did not identify any unmanaged external changes, in contrast to the 2016 report, in which 18.6% (*n* = 6) of EDs identified were related to unmanaged external changes. Although we did not observe any unmanaged external change EDs in this report, the widespread and prolonged closure of building water systems due to the coronavirus disease 2019 (COVID-19) pandemic might result in increased prevalence of this deficiency type.

Examination of the sources of EDs showed important differences in the origins of deficiencies between the WMP and no-WMP groups. Infrastructure and plumbed device failures were much more prevalent in facilities without WMPs (Figure 3). This divergence provides some evidence that WMPs are effective tools for identifying and mitigating *Legionella* risk in the plumbed components of a water system. It might also signal a shift in the types of risk that facilities encounter as they implement formal WMPs.

This is the first report categorizing EDs since CMS issued a memorandum [18] that required WMP implementation in certain types of healthcare facilities. WMPs rely on implementing actions to control the environmental conditions that permit the growth and spread of *Legionella*, such as monitoring temperature and disinfectant levels, recording completion of a flushing protocol for infrequently used pipes, and maintenance checks on essential equipment. To our knowledge, this is the first time that WMP deficiencies have been described in the literature. Nearly half of the investigation reports we reviewed (*n* = 6, 43%) originated in settings without a WMP. Two thirds of the outbreak investigations that took place in facilities without WMPs occurred in senior living facilities or hotels/resorts. Senior living facilities are an important target for WMPs because they serve a population that is especially vulnerable to LD.

This project is subject to several limitations. First, the sample is drawn from land-based, onsite outbreak investigations of LD in which CDC participated since 2015. The sample is limited in size and is not necessarily representative of all EDs or WMP deficiencies that can lead to outbreaks of LD. Second, the authors were limited to analyzing the information contained in each CDC investigation report. While there is a standard format for collecting and documenting pertinent details for outbreak investigations, there is inherent variation in the amount of detail from report to report. Additionally, our review does not control for differences in the size or underlying vulnerability of each facility’s population, which could have contributed to observed differences in numbers of LD cases and fatalities between facilities with and without WMPs. Causality between WMP status and clinical outcomes could not be assessed. Finally, the subjective nature of the abstraction process could have impacted our results. We attempted to address this concern by adhering to a detailed abstraction guide during reviews and relying on subject matter expert opinion in cases of disagreement regarding a classification.

Several steps should be taken to improve the utility and power of this abstraction methodology. First, the abstraction protocol should continue to be applied, refined, and improved by environmental and public health practitioners. Next, a standardized ED data collection tool should be developed to reduce the variability in environmental information collected during outbreak investigations. Additional data will be critical for evaluating the effectiveness of WMPs in preventing LD outbreaks and shaping the future of environmental prevention guidance.

We developed a flexible and widely applicable protocol to standardize the collection of information about EDs and WMP deficiencies from LD outbreak investigation reports. This protocol can be used by state and local health departments to build a robust dataset of environmental health information about contributing factors that lead to outbreaks of LD. Standardization of the ED and WMP-deficiency data collection process allows for cross-comparison of outbreak reports and prospective trend analysis. Trend analysis over time will be especially valuable in evaluating the effectiveness of WMPs, as well as identifying opportunities for improving prevention practices and WMP implementation.

Outbreaks of LD continue to pose a serious threat to public health. Nearly five years since ASHRAE’s release of industry standards to control and prevent *Legionella* exposures, requirements for implementing ASHRAE-compliant WMPs outside the healthcare setting are minimal. Our results suggest that use of ASHRAE-compliant WMPs in other settings (e.g., lodging and resort industries) where they are recommended could reduce the burden of LD. Environmental and public health professionals can help by incorporating the ASHRAE standard into building and public health codes, and emphasizing the importance of WMPs with non-traditional stakeholders such as industry organizations and risk managers. CDC has made tools available for public health professionals for setting up WMPs and investigating outbreaks of LD. They include the *Legionella* Environmental Assessment Form [24] and the *Legionella* Toolkit, [23] (both are available at www.cdc.gov/legionella). To maximize the effectiveness of existing WMPs, environmental health practitioners should reinforce the importance of verification, validation, documentation, and continuous program review steps of WMPs.

## Figures and Tables

**Table 1:** Case and environmental deficiency information identified in outbreaks of Legionnaires’ disease investigated by the Centers for Disease Control and Prevention (CDC), 2015–2019 (*n* = 14).

Environmental Deficiency Information				Case Information				
Year of Investigation	Setting	Water Management Program in Effect	Source of Environmental Deficiencies	Total	Confirmed Legionnaires’ Disease *	Confirmed Pontiac Fever *	Other ^†^	Number of Deaths
2015	Senior Living/LTCF ^‡^	No	• Human error • Plumbed device (cooling tower) • Plumbed device (hot tub) • Plumbed device (water tower) • Potable water (disinfectant) • Potable water (temperature) • Premise plumbing design	58	46	12	0	12
2016	Hotel/Resort	No	• Human error • Plumbed device (storage tank) • Plumbed device (hot tub) • Potable water (temperature) • Premise plumbing design	3	3	0	0	1
2016	Hospital	Yes	• Human error • Potable water (disinfectant) • Potable water (temperature) • Water management program	1	1	0	0	0
2016	Senior Living/LTCF	Yes	• Human error • Water management program	5	5	0	0	0
2017	Hospital	Yes	• Human error • Plumbed device (cooling tower) • Plumbed device (decorative fountain) • Potable water (disinfectant) • Potable water (temperature) • Water management program	4	4	0	0	0
2017	Hospital	Yes	• Potable water (disinfectant) • Potable water (temperature) • Water management program	3	3	0	0	0
2017	Senior Living/LTCF	Yes	• Human error • Water management program	6	6	0	0	1
2018	Senior Living/LTCF	No	N/A	2	2	0	2	1
2018	Senior Living/LTCF	Yes	• Human error	4	4	0	0	0
2018	Hotel/Resort	Yes	• Human error • Potable water (temperature) • Water management program	7	7	0	0	1
2018	Hotel/Resort	No	• Building design • Plumbed device (hot tub) • Potable water (temperature)	19	19	0	24	1
2018	Other	No	• Building design • Plumbed device (hot tub)	6	6	0	15	0
2019	Hospital	No	• Human error • Potable water (disinfectant) • Potable water (temperature) • Premise plumbing design	14	14	0	0	2
2019	Hospital	Yes	• Human error • Plumbed device (sink) • Potable water (temperature) • Water management program	5	5	0	0	2
			Total	137	125	12	41	21

* Confirmed Legionnaires’ disease and Pontiac fever cases were defined according to Council of State and Territorial Epidemiologists (CSTE) case definitions, available at: https://www.cdc.gov/legionella/health-depts/surv-reporting/case-definitions.html; ^†^ Includes suspected, possible, and probable cases of Legionnaires’ disease or Pontiac fever; for the purposes of this analysis, cases were defined using each outbreak’s case definitions. Note these classifications do not necessarily align with extant CSTE case definitions. ^‡^ Long-Term Care Facility.

## Data Availability

Restrictions apply to the availability of these data. Data were obtained from CDC and are available from the authors with the permission of CDC.

## Supplementary Materials

The following are available online at https://www.mdpi.com/2076-2607/9/1/89/s1, Abstraction Guide for Identifying Environmental Contributors to Outbreaks of Legionellosis, Table S1: Detailed descriptions of environmental deficiencies identified in outbreaks of Legionnaires’ disease investigated by CDC–United States, 2015–2019 (*n* = 14).
