# Supplementary material for: A Methodology for Classifying Root Causes of Outbreaks of Legionnaires’ Disease: Deficiencies in Environmental Control and Water Management
Source: Microorganisms. 2021 Jan 1;9(1):89. doi: 10.3390/microorganisms9010089 (PMC7824450; doi:10.3390/microorganisms9010089)
Supplement: Supplementary file 1 [file microorganisms-09-00089-s001.zip › Abstraction Guide.docx]

**Abstraction Guide for Identifying Environmental Contributors to Outbreaks of Legionellosis**

**How to use this Guide:**

- Please read the entire guide before conducting your first abstraction.
- [Sections I-III detail steps for conducting an abstraction of an outbreak investigation report.](#_Categorizing_Environmental_Deficien)
- [Section IV](#_Discerning_the_Relevance) provides guidance about determining which specific settings of a community cluster investigation should be included in an abstraction.
- [Section V](#_Tips_for_Coding) is a list of tips for coding certain ambiguous or confusing scenarios such as multiple references of a single environmental deficiency, coding the presence of biofilm, and *Legionella*-positive environmental samples.

This abstraction guide is intended to help classify different types of environmental information from *Legionella* outbreak investigation reports in a standardized format. The abstraction methodology is based on the environmental deficiency categorization method described by Garrison et al.^[[1]](#footnote-1)^ While the authors of this guide intend it to be useful in abstracting information from different styles of reports, users may find it is easiest to use the guide in conjunction with a report that includes a specific focus on the results of an [environmental assessment](https://www.cdc.gov/legionella/downloads/legionella-environmental-assessment.pdf) and/or an investigation of the root causes of an outbreak.

The goal of this methodology is to allow the detailed environmental and water management program deficiency information contained in Legionnaires’ disease outbreak investigative reports to be reported to environmental health professionals. This guide creates a set of rules for categorizing these types of deficiencies but does not include requirements for reporting. The specificity and amount of detail included in each report that uses this methodology is the responsibility of the authors. Authors should consider the importance of descriptive environmental information reported with the deficiencies they highlight to provide a complete account of the circumstances of the deficiencies identified. Clarifying rationale for classification decisions should be provided when appropriate. There may be instances in which contextual information that is not directly included in an investigation report helps clarify a classification decision. Authors are encouraged to include such information in their reports.

# Rules for Defining and Coding Environmental Deficiencies

**Definition of an Environmental Deficiency:** A maintenance or management deficiency that permits, or could permit, the presence, growth, spread, or aerosolized distribution of *Legionella* in or from a human-made water system.

1. **Ensure that the deficiency under consideration meets the definition of an environmental deficiency**.
2. **Ensure that the deficiency was identified first-hand by an on-site investigator. Do not code deficiencies identified previously by contractors or building staff.**
3. **Code the environmental deficiency using the appropriate deficiency category, as defined below:**
   - **Process Failure**: A specific process is missing or inadequate for preventing the growth or spread of *Legionella*.
   - **Human Error**: A specific person did not perform as expected.
   - **Equipment Failure**: A specific piece of equipment did not operate as expected or is inadequate for preventing the growth or spread of *Legionella*.
   - **Unmanaged External Change**: A specific adjustment not made to account for events outside a building water system.
4. **More than one deficiency category is allowed per environmental deficiency**.
   - Each environmental deficiency should be coded broadly, based on the descriptive information in the text. Code as many deficiency classifications as are clearly evident from the report for each environmental deficiency.

# Rules for Defining and Coding a Source

**Definition of the ‘Source’ field:** The system (generally, but not always a *physical* system) in which an environmental deficiency occurs. Remedying the specific inadequate condition in a source will resolve the environmental deficiency.

1. **Code the source of the deficiency**.
   - Ensure that the source reflects the system in which the deficiency is identified. A WMP may also be considered the source of a deficiency, **but only if the entity has an existing WMP.** An example of this scenario would be a WMP that is missing critical content such as a corrective action log or a water flow diagram.

**NOTE:** The source may be implied by the text.

1. **If the facility does not have a WMP in place, the source of an environmental deficiency can never be a water management program**. **Instead, code the source of the deficiency as the related physical system in which the deficiency was identified**.
   - To illustrate this rule, consider a deficiency of a lack of operation and maintenance records for a cooling tower. If the facility where this deficiency was identified had a WMP in place, the source of this deficiency could easily be classified as WMP, since proper recordkeeping is an essential part of a WMP. In the case where this deficiency was identified in a facility without a WMP in place, the source of this deficiency would default to the physical system in which the deficiency was identified—the cooling tower.
2. **A building management decision can be appropriately categorized as the source of an environmental deficiency**. These types of deficiencies should be classified as a “personnel error.” For example, a construction decision that creates a dead leg or a decision to use a residential-style hot tub in a commercial setting.

# Rules for Defining and Coding Water Management Program Deficiencies

1. **After all environmental deficiencies have been classified, return to each deficiency and assign it to a Water Management Program deficiency group, as defined below:**
   - **No WMP in place**: The facility lacks a WMP.

**NOTE:** There will be cases in which a report is not explicit regarding a WMP’s content inadequacy or improper implementation (e.g. a fixture was not flushed, but it is not clear from the report if a flushing protocol was included in the WMP). For these cases, determine the most appropriate WMP deficiency, record a justification, and note that further discussion is required. The final WMP deficiency category should be selected in consultation with subject matter experts (SMEs). Epidemiologists, industrial hygienists, and sanitarians with responsibility and experience in *Legionella* investigations can act in the SME role.

- - **Inadequate WMP Content**: One or more critical elements of the WMP are missing or insufficient as described by CDC’s *Legionella Toolkit*.
  - **Improper WMP Implementation**: Improper implementation of the WMP created conditions in which one or more environmental failures occurred.

1. **For investigation reports in which some buildings or portions of a facility or campus are covered by a WMP and others are not, the entire facility/campus should be considered to have a WMP in place.**
2. **More than one deficiency category is allowed per Water Management Program deficiency**.
   - Each WMP deficiency should be coded broadly, based on the descriptive information in the text. Code as many deficiency classifications as are clearly evident from the report for each WMP deficiency.

# Discerning the Relevance of Individual Entities to a Cluster Outbreak

Outbreak investigations are often complex and may involve several distinct entities. These individual entities could be organizations, businesses, or other types of facilities like schools or hospitals. It is common to suspect and investigate several different entities in the case of a community cluster investigation. **In such instances, abstractors should examine the epidemiological links to each individual entity investigated during the outbreak and exclude distinct entities that were not likely contributors to the outbreak**. An individual entity includes the entirety of the premises (land and buildings) associated with, or occupied by, a business or other organization when considered from a legal context.

For illustrative purposes, consider the following hypothetical outbreak investigation of a suburban gym with a hot tub:

An investigation identified a strong epidemiological link to a gym (5 of 6 cases in the community reported exposure to a gym hot tub). The sixth case did not report exposure to the gym but reported visiting a pharmacy across the street from the gym. In order to conduct a thorough investigation, the investigators scanned the area for a point source and found a cooling tower at a nearby hotel. A potential environmental deficiency was noted in the hotel’s cooling tower (lack of biocide). The investigation report notes that the cooling tower was not operational during the exposure period due to lack of cooling demand.

In theory, the gym’s hot tub and the hotel’s improperly maintained cooling tower meet the definition of an environmental deficiency as stated in section III. However, it is clear from the detail in the example that the cooling tower (not operational during the outbreak) was not a likely contributor to the outbreak. Therefore, the gym hot tub would be considered the most likely contributor to the outbreak, and only environmental deficiencies on the gym’s premises should be abstracted.

Abstractors should use their discretion when determining which facilities are likely contributors for each outbreak. It is possible that more than one entity could be implicated in an outbreak. In such cases, environmental deficiencies identified in each associated entity should be abstracted. Outbreak investigations that did not establish a convincing epidemiological link to any specific entity should not be abstracted.

# Tips for Coding Specific Scenarios

1. **Coding a single, general mention of a specific deficiency type vs. coding multiple, specific mentions of the same failure type**.
   - Code the single, more general mention of the deficiency when it implicates a deficiency with system-wide implications (like a water heater set at a temperature in the growth range) or pervasive temperature issues throughout a distribution system.
   - Code multiple, specific deficiencies when there is no system-level deficiency noted causing problems.
2. **Lack of clinical surveillance equipment is not an environmental deficiency**.
   - Example: Lack of on-site UAT capability
3. **Facilities that lack a Water Management Program can still have process failures**.
   - Example: Sub-optimal hot water holding temperature throughout a building.
4. **No Water Management Program in place is a standalone environmental deficiency, but only in cases when a facility is required to have a WMP in place as directed by the CMS Survey and Certification Memo QS O -17-30, VA Directive 1061, or other state and local laws and regulations.**
   - The environmental deficiency type should be classified as “process failure” and its source classified as “personnel error.”
5. **Positive environmental samples should not be coded as an environmental deficiency**.
   - They should be coded as supporting information of another directly related and identified deficiency. If there is no corresponding environmental deficiency, then do not code the positive environmental sample.
6. **Presence of biofilm/sediment.**
   - An observation of biofilm and/or sediment that does not indicate its cause should not be coded as an environmental deficiency on its own, unless it is identified in one of the following systems that include monitoring for or remediating biofilm as part of its standard maintenance procedures:
     1. cooling towers
     2. fountains
     3. water walls
     4. hot tubs
     5. ice machines
   - Biofilm/sediment should normally be classified *as a part* of a single environmental deficiency. For example:
     1. Biofilm/sediment observed due to lack of regular hose maintenance or sink aerator replacement is part of a process failure
     2. Biofilm/sediment observed related to an unmanaged external change (e.g., nearby construction)
   - Observations of biofilm or sediment that do not note a cause or origin should NOT be coded as an environmental deficiency, because it cannot be discerned what type of deficiency led to the film/sediment formation. For example:
     1. Biofilm/sediment in an old galvanized pipe plumbing system
7. **Do not code permissive temperatures that occur in a cold-water distribution system. This may be unavoidable in certain parts of the country due to seasonal temperature fluctuations.**
8. **Classify the source of deficient water parameters in potable water using “potable water” as the source.** Examples of such deficiencies are:
   - Faucet water temperatures in the *Legionella* growth zone
   - Shower water has inadequate disinfectant level

*Rationale*: These are deficient aspects of the potable water system that could have several causes that may be difficult to identify based on the information in a report (high ambient temperature, water heater set below optimal temperature, increased water age, etc.).

1. **Classify the source of deficient water parameters in non-potable water using “plumbed device” as the source (all non-potable water parameters are reported in reference to plumbed devices).**

Examples of such deficiencies are:

- - Hot tub in use without proper disinfectant
  - Lack of corrosion inhibitor in cooling tower feed water

*Rationale*: These are deficient aspects of a water system that relates uniquely to the operation of the plumbed device, which is separate from normal potable water characteristics.

**Note: The circumstances of every investigation are unique. Abstractors and subject matter experts should use their best judgement to classify environmental deficiencies as accurately as possible, and to reflect the reality of the findings on-site.**

1. Garrison L, et al. Vital Signs: Deficiencies in Environmental Control Identified in Outbreaks of Legionnaires’ Disease—North America, 2000-2014. *MMWR* 2016 Jun 10;65(22):576-84. [↑](#footnote-ref-1)
